# Trends in Breast Cancer Stage and Mortality in Michigan (1992–2009) by Race, Socioeconomic Status, and Area Healthcare Resources

**DOI:** 10.1371/journal.pone.0061879

**Published:** 2013-04-29

**Authors:** Tomi F. Akinyemiju, Amr S. Soliman, Glenn Copeland, Mousumi Banerjee, Kendra Schwartz, Sofia D. Merajver

**Affiliations:** 1 Department of Epidemiology, Columbia University Mailman School of Public Health, New York, New York, United States of America; 2 Department of Epidemiology, University of Nebraska School of Public Health, Omaha, Nebraska, United States of America; 3 Michigan Cancer Surveillance Program, Michigan Department of Community Health, Lansing, Michigan, United States of America; 4 Department of Biostatistics, University of Michigan School of Public Health, Ann Arbor, Michigan, United States of America; 5 Department of Family Medicine and Public Health Sciences and Barbara Ann Karmanos Institute, Wayne State University School of Medicine, Detroit, Michigan, United States of America; 6 Department of Internal Medicine, University of Michigan Medical School, Ann Arbor, Michigan, United States of America; 7 University of Michigan Center for Global Health, Ann Arbor, Michigan, United States of America; Baylor College of Medicine, United States of America

## Abstract

The long-term effect of socioeconomic status (SES) and healthcare resources availability (HCA) on breast cancer stage of presentation and mortality rates among patients in Michigan is unclear. Using data from the Michigan Department of Community Health (MDCH) between 1992 and 2009, we calculated annual proportions of late-stage diagnosis and age-adjusted breast cancer mortality rates by race and zip code in Michigan. SES and HCA were defined at the zip-code level. Joinpoint regression was used to compare the Average Annual Percent Change (AAPC) in the median zip-code level percent late stage diagnosis and mortality rate for blacks and whites and for each level of SES and HCA. Between 1992 and 2009, the proportion of late stage diagnosis increased among white women [AAPC = 1.0 (0.4, 1.6)], but was statistically unchanged among black women [AAPC = −0.5 (−1.9, 0.8)]. The breast cancer mortality rate declined among whites [AAPC = −1.3% (−1.8,−0.8)], but remained statistically unchanged among blacks [AAPC = −0.3% (−0.3, 1.0)]. In all SES and HCA area types, disparities in percent late stage between blacks and whites appeared to narrow over time, while the differences in breast cancer mortality rates between blacks and whites appeared to increase over time.

## Introduction

Overall age-standardized breast cancer mortality rates have declined significantly over time in the U.S [1]–[7] and in Michigan [8]. However, widening disparities in breast cancer mortality between black and white patients has been consistently reported across the US and within the state of Michigan [4], [9]–[12]. According to Surveillance Epidemiology and End Results (SEER) data, breast cancer mortality rates declined from 35 per 100,000 to 29 per 100,000 for black women and from 30 per 100,000 to 21 per 100,000 for white women between 1990 and 2000 [4]. In Michigan, the breast cancer mortality rate was 23.4 per 100,000 for whites and 33.8 per 100,000 among blacks between 2003 and 2007 [12]. In addition, the National Center for Health Statistics reported that between 1999 and 2007, black women were more likely than any other racial group in the US to die of breast cancer [13].

Possible reasons for the observed racial disparity in mortality include differential access to breast cancer screening and timely diagnosis at early stages, as well as adequate treatment [11], [14]. Adequate access to screening, diagnosis and treatment were found to be associated with socio-economic status (SES), both at the individual and area level [15]–[18]. For instance, breast cancer mortality rates in least deprived U.S. counties declined at a higher rate compared with rates in counties that were most deprived [15]. Individuals with higher SES have better breast cancer outcomes compared with those with lower SES, even though racial disparities were still present [19].

In addition, lack of availability of healthcare resources (HCA) has been associated with poorer outcomes in many aspects of breast cancer including screening [20], diagnostic follow-up [21], [22], stage of presentation [23], [24], treatment [25], [26] and survival [27]. Despite these improvements in our understanding of the contributors to racial disparities in breast cancer mortality, more research is needed to understand long term trends in these factors that influence the racial disparities of breast cancer outcomes. Recent studies using national SEER data have reported significant racial and county level socio-economic disparities in breast cancer screening and mortality trends [15], [28], [29]. We sought to assess the presence of racial and socio-economic as well as HCA disparities on breast cancer stage of presentation and mortality in Michigan.

Access to healthcare is a complex, multidimensional concept that is difficult to distill down to a single measure. It has been described as encompassing dimensions of availability, accessibility, affordability and acceptability [30]. Other researchers have further defined access to healthcare as being a function of enabling factors, both individual (age, race, income) and neighborhood (number of physicians, hospitals or mammography centers) [31]–[33]. For this study, we chose to focus on the aspect of healthcare availability as a predictor of breast cancer mortality at the area level.

Although racial disparities in breast cancer mortality have been reported for a while, we were interested in examining the recent trends to assess if the disparities appear to be increasing or decreasing. Furthermore, racial disparities by geography have been well reported especially since African-Americans and Hispanics tend to live in different areas than whites. However, few studies have examined black and white women residing in similar areas to see if they have similar breast cancer mortality experiences over time. We were interested comparing women residing in relatively homogenous areas (zip-code level), further stratified with regards to SES and HCA.

Several studies have shown that study results can vary significantly depending on which geographic level neighborhood variables are measured [34]–[37]. For this study, the zip code level was chosen as the ideal level because we believe the census tract level may be too small geographically to provide a meaningful measure of healthcare resources available to women. The aim of this study was to assess the influence of zip-code level HCA and SES on breast cancer stage at diagnosis and mortality trends for white and black patients in Michigan between 1992 and 2009.

## Methods

### Study Population

Annual breast cancer stage of diagnosis and mortality data were obtained from the Michigan Cancer Surveillance Program (MCSP) at the Michigan Department of Community Health. The MCSP is a member of the North American Association of Central Cancer Registries and is certified for its quality and high level of data completeness. The data included breast cancer deaths for patients in the age groups of 20 years and older separately for blacks and whites in each zip-code in Michigan and for every year between 1992 and 2009. The stage of diagnosis contained the proportion of late stage diagnoses separately for blacks and whites per zip code for each year between 1992 and 2008. Late stage of diagnosis was defined as regional and distant breast cancer according to the 2000 SEER summary stage criteria. Annual age-adjusted mortality rates by race and zip code were calculated using the 2000 U.S standard population. IRB approval for use of the data was obtained from the University of Michigan as well as the Michigan Department of Community health.

### Study Variables

Since the analytic dataset consisted of data from 1992 to 2008, we chose the year 2000 as a mid-point of the interval for which to define zip-code level SES and HCA. To ensure that our measure of area-SES was stable, we compared zip-code level SES scores for 1990 and 2000 and found them to be highly correlated (Correlation Coefficient = 0.94, p<0.05). Therefore, we assumed that the distribution of zip-code level variables used in our analysis was similar throughout the study period.

#### Socio-Economic status

**Figure 1:**
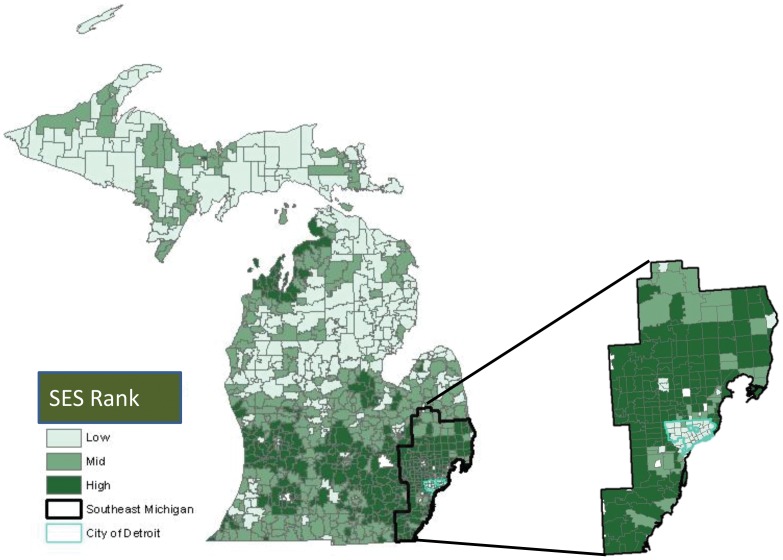
Geographical Distribution of Socio-economic Status among Michigan zip-codes.

We constructed a measure of zip-code level SES by using principal components analysis (PCA) using data obtained from the US Census Bureau [38]. Four variables that we believe are most closely related to SES based on previous studies were subject to PCA; a) proportion of adults ages 25 and older with over 4 years of education, b) proportion of residents ages 16 and older in the labor force but unemployed, c) proportion of households in poverty and d) the median household income [39], [40]. The first principal component accounted for 62% of the variance in the dataset, and was retained for further analysis as a measure of zip-code level SES. SES was categorized into tertiles corresponding to low, middle and high SES. The geographic distribution of SES among Michigan zip-codes is presented in Figure 1.

#### Healthcare resources

**Figure 2:**
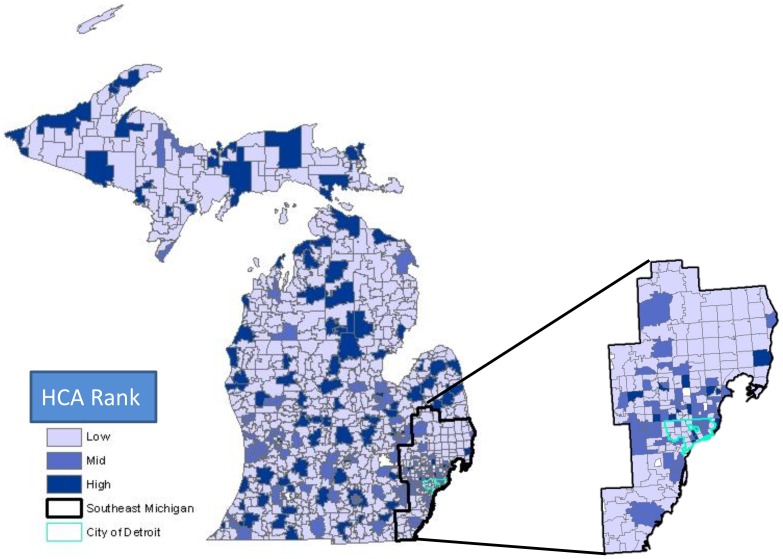
Geographical Distribution of Area Healthcare Resources among Michigan zip-codes.

We sought to define zip code level HCA as the availability of healthcare personnel and facilities in Michigan. However, data on healthcare personnel such as the count of doctors and nurses at the zip-code level for 2000 in Michigan was not available. Therefore, we defined zip-code level HCA as the number of hospitals and mammography facilities available in each Michigan zip-code. A list of names and addresses of all licensed hospitals in Michigan in 1999 were obtained from the Division of Licensing and Certification at the Michigan Bureau of Health Systems. In addition, a list of names and addresses of all licensed mammography facilities operational in Michigan in 2000 were obtained from the Michigan State Radiation Safety Section. For our final analysis, we defined zip-level HCA as the sum of the number of hospitals and mammography facilities divided by the zip-code population in 2000, multiplied by 10,000. HCA was categorized into tertiles; low, middle and high. The geographic distribution of HCA among Michigan zip codes is presented in Figure 2.

### Analytic Methods

Age-adjusted breast cancer mortality rate by race was calculated for all zip codes in Michigan annually for 1992–2009, and linked with the dataset containing zip-code level SES and HCA. Annual median age-adjusted breast cancer mortality rates by race, SES and HCA were calculated. Median percent late stage diagnosis per zip code was also calculated by race, SES and HCA. In addition, Joinpoint regression with a maximum of three joinpoints was used to compare changes in the median zip-code level mortality rate and percent late stage diagnosis over time by race, SES and HCA. Joinpoints are used to test for significant changes in the slope or trend, and also to assess if two regression functions are coincident or parallel (Joinpoint, Version 3.5.1). Trends in median age-adjusted mortality and percent late stage by race, SES and HCA are defined as increasing or decreasing when the Annual Percent Change is statistically significant (p<0.05), and stable if otherwise. Significant trends throughout the entire years of analysis are also assessed by the Average Annual Percent Change (AAPC). Furthermore Poisson regression was used to model the temporal trend in annual median mortality rate and percent late stage by race adjusting for area SES and HCA.

## Results

### Percent Late Stage Diagnosis

Between 1992 and 2008, 27% of black patients and 21% of white patients were diagnosed at a late stage (Table 1). Median percent late stage diagnosis declined as zip-code SES increased for both black and white patients. The median percent late stage diagnosis for black patients was 33%, 17% and 11% for low, middle and high SES zip-codes respectively. Among white patients the median proportion of late stage diagnosis was 23%, 21%, 20% for low, middle and high SES zip-codes respectively (Table 1). Median percent late stage diagnosis for black patients was 29%, 26% and 27% for low, middle and high HCA zip-codes respectively. Among white patients, the median percent late stage of diagnosis was 20%, 25% and 23% for low, middle and high HCA zip-codes respectively (Table 1).

**Table 1:** Distribution of Breast Cancer Mortality and Percent Late Stage by Zip-Code Characteristics.

	Frequency % (n)		Median Mortality Rate^a^		% Late Stage^b^	
	Black	White	Black	White	Black	White
**Total**	13.3 (3,684)	86.7 (20,241)	32.07	28.80	27.27	21.05
**SES**						
Low	76.1 (2,804)	17.6 (3,572)	27.99	38.35	33.33	23.08
Middle	11.5 (422)	29.9 (6,055)	40.63	31.74	16.67	20.83
High	11.7 (431)	51.3 (10,390)	72.15	25.47	10.79	20.00
**HCA**						
Low	57.1 (2102)	48.3 (9776)	31.63	34.19	28.57	20.00
Middle	32.6 (1201)	34.9 (7079)	32.23	21.79	26.12	25.00
High	9.6 (355)	15.7 (3171)	33.95	25.89	26.97	23.08

a Median of zip-code and year specific mortality rates by race, 1992–2009.

b Median of zip-code and year specific proportion of late stage by race, 1992–2008.

**Figure 3:**
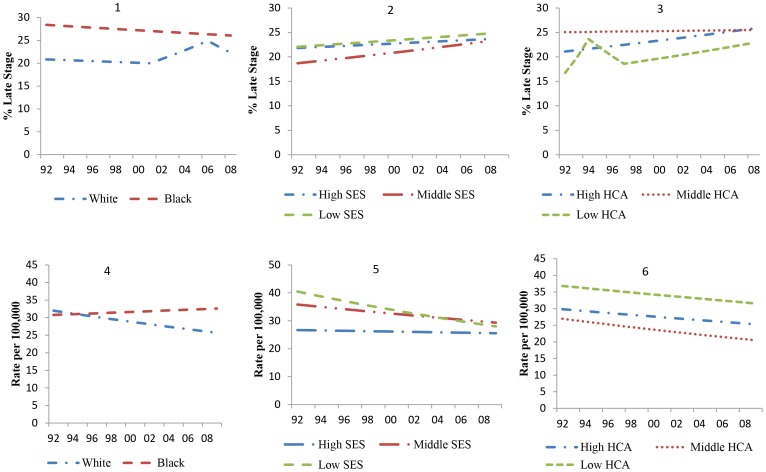
Median Predicted Zip-Code Percent Late Stage Diagnosis (1992–2008) and Age-Adjusted Mortality Rate (1992–2009) by Race, SES and HCA.

Black patients were consistently diagnosed at a late stage more frequently than white patients over time (Figure 3–1). However, there was a statistically significant increase of 4.5% (CI: 0.4, 8.7) in late stage diagnosis among white patients between 2002 and 2006 (Table 2). There were also apparent differences in late stage of diagnosis between SES groups (Figure 3–2). Late stage diagnosis among the low SES group remained stable over the study period, but increased in the middle and high SES groups (Table 2). Clear gradients also existed between HCA groups (Figure 3–3). The high HCA group experienced a statistically significant increase which narrowed the gap between middle and high access groups by 2008 (Table 2). The low HCA group experienced several statistically significant trends, although remained lower than other groups.

**Table 2:** Trends^†^ in Percent Late Stage (1992–2008) and Median Mortality Rate (1992–2009) by Race, SES and HCA.

	Trend 1		Trend 2			Trend 3			Average Annual Percentage Change
	Years	APC (95% CI)	Years		APC (95% CI)	Years	APC (95% CI)		
**Percent Late Stage**									
**Race**									
Black	1992–1995	−7.9 (−17.9,3.4)		1995–2002	2.0 (−1.9,6.1)	2002–2008		−2.4 (−6.1,1.5)	−0.5 (−1.9, 0.8)
White	1992–2001	−0.4 (−1.6,0.7)		2001–2006	4.5 (0.4–8.7)*	2006–2008		−5.3 (−16.5,7.4)	1.0 (0.4,1.6)*
**SES**									
Low	1992–2002	−0.0 (−2.1,2.1)		2002–2006	5.2 (−8.0,20.2)	2006–2008		−10.1 (−31.2,17.4)	0.7 (−0.2,1.7)
Middle	1992–1994	17.2 (−11.6,55.4)		1994–1997	−7.0 (−29.9,23.2)	1997–2008		2.5 (0.5,4.4)*	1.4 (0.2,2.6)*
High	1992–1997	−2.8 (−6.7,1.2)		1997–2008	1.6 (0.4,2.9)*				1.6 (0.4,2.9)*
**HCA**									
Low	1992–1994	18.9 (0.9,40.0)*		1994–1997	−7.7 (−21.6,8.7)*	1997–2008		1.9 (0.8,3.0)*	1.9 (0.8,3.0)*
Middle	1992–1999	−1.6 (−3.2,0.2)		1999–2002	2.7 (−9.8,16.8)	2002–2008		0.2 (−2.0,2.4)	0.1 (−0.5,0.7)
High	1992–1994	−10.3 (−37.5,28.7)		1994–2008	2.0 (0.3,3.7)*				2.0 (0.3,3.7)*
**Mortality Rate**									
**Race**									
Black	1992–2000	−0.2 (−2.6,2.3)		2000–2007	1.5 (−2.3,5.5)	2007–2009		−6.0 (−25.1,18.0)	−0.3 (−0.3,1.0)
White	1992–1995	1.4 (−7.4,11.1)		1995–1998	−2.8 (−18.9–16.7)	1998–2009		−1.2 (−2.4, −0.0)*	−1.3 (−1.8, −0.8)*
**SES**									
Low	1992–2002	−2.6 (−4.6, −0.6)*		2002–2006	−0.7 (−12.9,13.1)	2006–2009		−2.9 (−14.8,10.6)	−2.1 (−2.9, −1.3)*
Middle	1992–2004	−1.4 (−2.5, −0.3)*		2004–2007	1.5 (−15.9,22.5)	2007–2009		−5.7 (−21.9,13.8)	−1.2 (−1.7, −0.7)*
High	1992–1994	3.5 (−8.0,16.3)		1994–2002	−0.0 (−1.6,1.6)	2002–2009		−1.1 (−2.7,0.4)	−0.3 (−0.8,0.3)
**HCA**									
Low	1992–1995	0.5 (−7.7,9.4)		1995–2009	−1.1 (−1.8, −0.3)*				−0.9 (−1.4, −0.4)*
Middle	1992–2001	−2.0 (−3.9, −0.1)*		2001–2007	−0.4 (−5.0,4.5)	2007–2009		−6.8 (−24.7,15.4)	−1.6 (−2.2, −1.0)*
High	1992–2003	−1.9 (−3.6, −0.2)*		2003–2007	3.2 (−9.2,17.1)	2007–2009		−7.3 (−28.1,19.6)	−1.0 (−1.7, −0.2)*

* P<0.05; APC Annual Percent Change; CI Confidence Interval; ^†^Trend years may include different time periods based on Joinpoint regression modeling.

**Figure 4:**
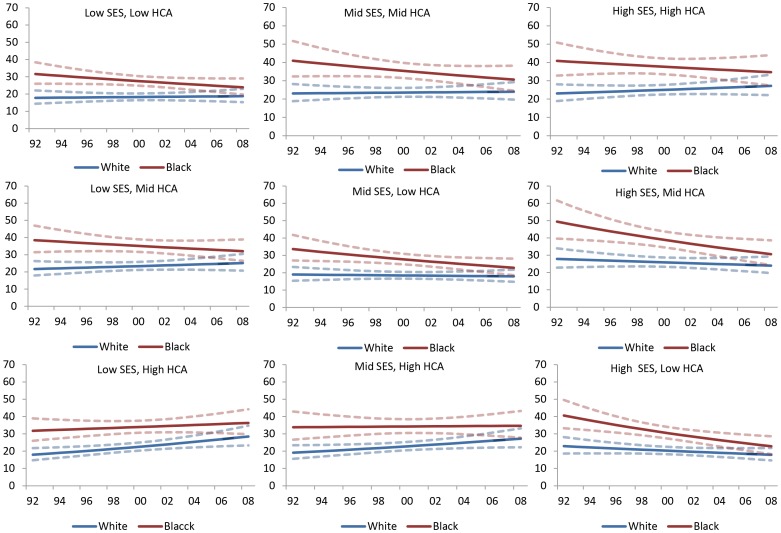
Median Predicted Zip-Code Percent Late Stage Diagnosis (1992–2008) by Race and Neighborhood Type.

Significant disparities in late stage diagnosis were also observed between blacks and whites when examined within strata of zip-code level SES and HCA (Figure 4). Blacks were more likely to be diagnosed at a late stage in each of the area types. In most area types except low SES/high HCA and mid SES/high HCA, the percentage of late stage diagnosis among blacks declined over time. In contrast, among white patients the percentage of late stage diagnosis increased over time in most area types, excluding mid SES/low HCA, high SES/mid HCA and high SES/low HCA. In all area types, differences in percent late stage between blacks and whites appear to have narrowed over time.

### Age-Adjusted Mortality Rates

Between 1992 and 2009, 3,684 black patients and 20,241 white patients died of breast cancer in Michigan (Table 1). Age-adjusted breast cancer mortality rate during this period was 32 per 100,000 among black patients and 29 per 100,000 among white patients. Among black patients, median age-adjusted breast cancer mortality rate increased as zip-code level SES increased. The median age-adjusted mortality rate was 28 per 100,000, 41 per 100,000 and 72 per 100,000 for low, middle and high SES zip-codes respectively. Among white patients, the reverse trend was observed; the median age-adjusted mortality rate was 38 per 100,000, 32 per 100,000 and 25 per 100,000 for low, middle and high SES zip-codes respectively. As zip-code level HCA increased, median age-adjusted mortality rate increased for black patients; 32 per 100,000, 32 per 100,000 and 34 per 100,000 for low, middle and high HCA zip-codes respectively. Among white patients, the median age-adjusted mortality rate was 34 per 100,000, 22 per 100,000 and 26 per 100,000 for low, middle and high HCA zip-codes respectively.

**Figure 5:**
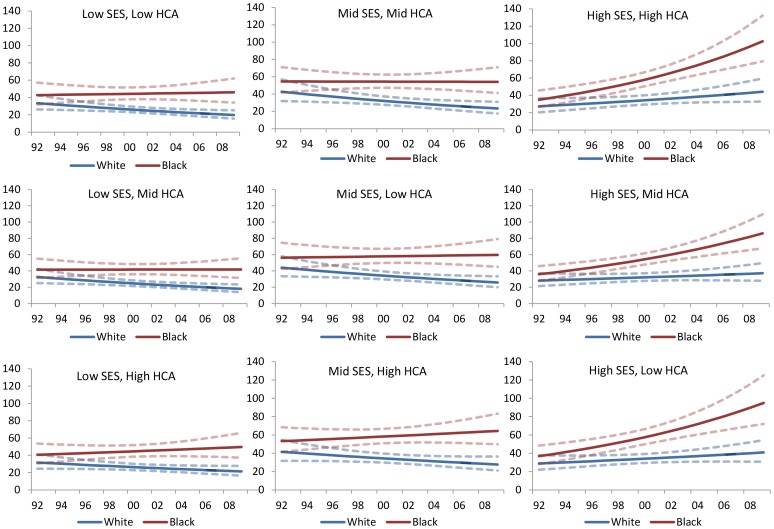
Median Predicted Zip Code Age-Adjusted Mortality Rate (1992–2008) by Race and Neighborhood Type.

Between 1992 and 2009, the age-adjusted breast cancer mortality rate appeared to have declined among whites, but remained statistically unchanged among blacks, resulting in higher rates among blacks patients compared with whites (Figure 3–4). There were no joinpoints in median mortality rates for blacks, however whites observed a statistically significant change of −1.3% (CI: −1.8, −0.8) during the study period (Table 2). There were initial large differences in rates between SES levels in 1992 with the highest rates among low and middle SES groups but these appeared to have narrowed significantly by 2006 (Figure 3–5). This trend is reflected in the statistically significant joinpoints in median mortality rates for low and middle SES groups (Table 2). There are also clear gradients between the three HCA groups; low HCA groups had the highest mortality rate, followed by high HCA and middle HCA groups (Figure 3–6). All three HCA groups had statistically significant joinpoints that suggested declining rates over time. Each of the HCA groups were statistically different from each other (i.e. not coincident), but were parallel to each other.

In all area types, blacks had higher mortality rates compared with whites, and the disparity between the two racial groups appeared to have increased over time (Figure 5). Among black patients, the age-adjusted mortality rate showed marked increases in all area types except mid SES/mid HCA. The largest increase appeared to be in high SES areas, regardless of HCA. Among white patients, annual age-adjusted mortality rates declined in all area types except high SES/high HCA, high SES/mid HCA and high SES/low HCA. The largest increase in mortality rates among black women also was observed in high SES areas, regardless of HCA. In all area types, differences in age-adjusted breast cancer mortality rates between blacks and whites appear to have increased over time.

## Discussion

The purpose of this study was to assess trends in late stage diagnosis and breast cancer mortality rate among black and white women in Michigan between 1992 and 2009, accounting for area SES and HCA. Our findings suggest that in the past 18 years, black and white breast cancer patients in Michigan have had different experiences with the disease based, in part, on their area of residence. In the period between 1992 and 2008, when both racial groups are initially examined without accounting for area type, the proportion of late stage presentation increased annually by 1.0% among white patients, but remained statistically unchanged among black patients. In the same period, the breast cancer mortality rate declined annually by −1.3% among white patients but remained statistically unchanged among black patients.

Further examination of the trends based only on area SES characteristics suggests that the proportion of late stage presentation narrowed between SES groups by 2008. This narrowing trend was due to the increase in the proportion of late stage presentation among the middle and high SES groups, compared with the statistically unchanged trend among the low SES groups. Similarly, a narrowing of the mortality rate trend occurred between 1992 and 2009; although mortality rates in the high SES group remained statistically unchanged, the low and middle SES groups experienced significant declines in mortality rates irrespective of race. This trend resulted in limited disparity in breast cancer mortality rates between low, middle and high SES groups by 2009, irrespective of race. When outcomes were further examined based only on area HCA, surprisingly, the proportion of late stage presentation appeared lower among the low HCA group and highest among the middle HCA group. However, by 2008, the low and high HCA groups had experienced significant increases, while the middle HCA remained statistically unchanged. This trend resulted in similarly higher rates for high and middle HCA groups compared with the low HCA groups at the end of the period. Mortality rates declined for all HCA groups; however, disparities remained between the groups at the end of the study period irrespective of race.

Additionally, examining late stage diagnosis and mortality rate trends between black and white patients from similar area types suggests that large disparities still remained at the end of the study period. Black patients were consistently diagnosed at a late stage more frequently than whites, although they have experienced a significant reduction in the proportion of late stage diagnosis over time. White patients, on the other hand, experienced significant increases in the proportion of late stage diagnosis in all area types. The reverse scenario is observed in age-adjusted mortality rates. Black patients had higher mortality rates than white patients in all area types, and they have observed a significant increase in mortality rates over time. The largest annual increase appeared to have been among black patents in the high SES/high HCA group; this group had the smallest amount of disparity between black and white patients at the beginning of the study period. White patients in the high SES/high HCA group also experienced a large increase in mortality rate during the study period.

Overall across all the study years, the highest proportion of late stage diagnoses was observed among black patients, patients residing in low SES zip-codes and patients residing in middle HCA zip-codes. The groups that had the highest breast cancer mortality rates were black patients, patients residing in low SES zip-codes and patients residing in low HCA zip-codes. These findings are similar to others that have found consistent disparities in cancer outcomes by race, socio-economic status and HCA [28]. In addition, other studies have reported the correlation between late stage at diagnosis and high mortality rates, especially among blacks [10], [41]. This correlation has been attributed to the lower likelihood of receiving appropriate treatment, and a higher likelihood of co-morbid conditions such as diabetes and hypertension [42]–[44]. Other studies suggest that aggressive, triple-negative breast cancer sub-types may be responsible for the higher mortality rates observed among blacks [45]. These cases account for less than 25% of all invasive breast cancer cases, with higher rates observed among blacks compared with whites [46]. Therefore, research efforts should be focused on understanding factors such as SES and HCA that affect the vast majority of breast cancer patients of all races, but may also be related to the development of aggressive breast cancer sub-types [47].

The trend of higher mortality rates in higher SES areas has been observed previously at the county level [15]. Possible explanations for the high percent late stage diagnosis and age-adjusted mortality rate among black patients even in high SES and HCA zip-codes could be attributed to other dimensions of access to healthcare not captured by availability of healthcare resources. Cultural or language barriers as well as historical mistrust of the medical system could potentially limit the ability of black women to benefit from available healthcare resources. These factors have been consistently associated with reduced contact with healthcare facilities among minority populations in the US [48], [49].

Furthermore, residing in a high SES zip-code may not necessarily mean higher SES individually. Lack of reliable transportation, time off from work or health insurance may be major factors that could preclude routine use of healthcare facilities. Furthermore, research studies have suggested that retaining social and family networks are a major reason why black women reside in low SES areas [50]. Consequently, residing in a high SES area may result in the loss of established social networks that have also been shown to be important to improved psychosocial wellbeing and health outcomes [49], [51], [52]. In addition, other studies have shown that large disparities exist in disease outcomes including mortality between blacks and whites, and these disparities are sometimes largest in high SES neighborhoods [53], [54]. This may be due to reasons such as psychosocial stress, perceived racism, or social isolation.

Another possible reason for the high mortality rates observed in high SES and HCA zip-codes is out-migration of low risk individuals from these areas, however this migration would have to be differential based on risk. That is, there would have to be a selection factor that made low risk individuals more likely to move out compared to high risk individuals. It is difficult to test this empirically since we do not have access to data on breast cancer risk factors for this study. In addition, if such high SES zip-codes also had better healthcare facilities, it is conceivable that in-migration of high risk individuals (or those already diagnosed breast cancer cases) would also increase observed mortality rates.

Among white patients in Michigan, the observed increase in the proportion of late stage diagnosis is surprising, and further studies are warranted to replicate this finding and to offer potential reasons. This may be a finding unique to Michigan due to the severe economic downturn experienced in the state. This could have resulted in loss of employment, loss of insurance coverage for individuals and families, potentially resulting in less access to healthcare resources and lower cancer screening rates. We were unable to test this empirically in this study, and further research in this area is warranted. In addition, we cannot discount the possibility of an increase in aggressive sub-types of breast cancer among white women which progresses very rapidly and may not always be detected by annual mammography screening. However, more research is also needed in this area.

Among white women residing in high SES neighborhoods, mortality rates appeared to increase. It is reasonable to expect that inconsistencies in breast cancer medication use (which can be quite expensive) may lead to higher mortality in this group. We believe that in high SES areas, it is possible for a family to lose a source of income but still make enough to be disqualified from programs focused on cancer screening and treatment for low income women e.g. the Michigan Breast and Cervical Cancer Control program. In addition, women who have been covered by health insurance for a long period of time may be unaware that such services even existed. This same reasoning may apply to the increase in proportion of late stage diagnosis observed in middle and high SES areas; lack of familiarity with programs that have traditionally been designed to assist low income women may have put women residing in middle and high SES areas at a disadvantage compared with women residing in low SES areas. These are potential areas for future research studies.

A major strength of this study is the measure of area characteristics at the zip-code level, a smaller geographic area than the county which may provide a more homogenous population with respect to SES and HCA. Most research studies use county level characteristics in assessing SES disparities in breast cancer [15], [28], [29], [32], [55]–[57], and none has assessed zip-code level characteristics in relation to breast cancer in Michigan. In addition, the availability of data on area SES and HCA improved our ability to parse out differences in late stage and mortality trends between black and white women residing in similar areas. There are inconsistencies in the literature about at level at which to measure area level variables, however we believe that it is important to choose a geographic level that makes sense for the disease entity being studied. Since breast cancer is a relatively rare disease, and healthcare resources are not likely concentrated at very small levels such as the census tract, we believe that the use of zip codes as our geographic unit is ideal for our research aim.

There are some limitations to this study. First, HCA was defined based only on the availability of healthcare facilities at the zip-code level. The availability of healthcare personnel could have improved this measure, but lack of data precluded its inclusion in this analysis. However, facilities and personnel are likely highly correlated at the area level i.e. the more facilities available, the more personnel will be present. In addition, zip-code level measures of SES and HCA were used in this study as indirect measures; we did not account for the potential porosity of geographic boundaries in which residents of low HCA zip codes may have access to and chose to travel to high HCA zip-codes for healthcare. Furthermore, zip codes that did not exist during the 2000 census were dropped from the analysis, potentially introducing some bias if these zip codes were significantly different from the rest of the state in terms of our outcome of interest.

In summary, future studies in the US should focus on better understanding the factors contributing to the rise and fall of proportion of late stage at diagnosis and mortality rate trend over time through detailed analysis of trends in risk factors known to be associated with these outcomes. In addition, in-depth clinical studies are needed to explore the possibility of rising aggressive sub-types of breast cancer among white women. These aggressive cancers may be partly responsible for rising late stage diagnosis among white women, and understanding this sub-group may help researchers in developing guidelines for more frequent screening in this population. This study and future research will be very helpful in better understanding population sub-groups that are disproportionately experiencing adverse outcomes despite improvements in screening and treatment regimens. It will be very helpful to examine trends in other regions of the US that may have experienced significant economic downturn to assess the impact of loss of employment and insurance on breast cancer stage at presentation and mortality. Globally, as breast cancer rates are projected to increase, it will be important to conduct studies like these to identify vulnerable sub-groups that may benefit from concentrated resources aimed at increasing access to timely screening and adequate treatment.
